# Censusing marine eukaryotic diversity in the twenty-first century

**DOI:** 10.1098/rstb.2015.0331

**Published:** 2016-09-05

**Authors:** Matthieu Leray, Nancy Knowlton

**Affiliations:** National Museum of Natural History, Smithsonian Institution, Washington, DC 20013, USA

**Keywords:** meiofauna, protist, plankton, metabarcoding, 18S

## Abstract

The ocean constitutes one of the vastest and richest biomes on our planet. Most recent estimations, all based on indirect approaches, suggest that there are millions of marine eukaryotic species. Moreover, a large majority of these are small (less than 1 mm), cryptic and still unknown to science. However, this knowledge gap, caused by the lack of diagnostic morphological features in small organisms and the limited sampling of the global ocean, is currently being filled, thanks to new DNA-based approaches. The molecular technique of PCR amplification of homologous gene regions combined with high-throughput sequencing, routinely used to census unculturable prokaryotes, is now also being used to characterize whole communities of marine eukaryotes. Here, we review how this methodological advancement has helped to better quantify the magnitude and patterns of marine eukaryotic diversity, with an emphasis on taxonomic groups previously largely overlooked. We then discuss obstacles remaining to achieve a global understanding of marine eukaryotic diversity. In particular, we argue that 18S variable regions do not provide sufficient taxonomic resolution to census marine life, and suggest combining broad eukaryotic surveys targeting the 18S rRNA region with more taxon-focused analyses of hypervariable regions to improve our understanding of the diversity of species, the functional units of marine ecosystems.

This article is part of the themed issue ‘From DNA barcodes to biomes’.

## 1. State of knowledge based on traditional taxonomy

Human activities are having an increasing effect on ocean biodiversity, although it remains the case that relatively few marine species are reported to have gone extinct [1]. However, impact assessments and monitoring initiatives are based almost exclusively on large and conspicuous species that represent a minor fraction of marine diversity. Small (less than 2 mm) and cryptic organisms, which play important ecological roles despite being inconspicuous, remain overlooked in biodiversity surveys [2]. This highlights a major limitation in our ability to monitor biological communities: How can we establish biological baselines, quantify changes in biodiversity over time and understand the consequences of community shifts on ecosystem services if most species are unknown to science or cannot be easily surveyed?

Estimates of the number of existing marine species, all based on indirect approaches (e.g. experts' opinion [3] and extrapolations from rates of species description [4], rates of discovery of higher taxa [5] or known areas or faunas [6]), range from 0.3 to more than 10 million species. According to the World Register of Marine Species (WORMS, [7]), there were 228 739 accepted eukaryotic marine species as of September 2015 (of which Animalia constituted 195 702 species, Plantae 9689 species, Chromista 21 403 species, Protozoa 589 species and Fungi 1356 species). This suggests that between 24 and 98% of all marine eukaryotic species remain to be described, with the proportion of unknown diversity varying greatly among groups. Taxonomic experts have estimated that fewer than 10% of species might be formally described in the most cryptic taxonomic groups (e.g. isopods, micro-gastropods, nematodes, copepods and some Chromista [3]). Even among well-known groups such as marine mammals, new species continue to be discovered [8]. For many groups, the absence of diagnostic morphological characters (e.g. nematodes [9]), the lack of taxonomic expertise and the time required to describe or identify species [10] have been major impediments to obtaining a comprehensive understanding of marine diversity. When coupled with limited sampling, particularly in some regions of very high diversity and endemicity [11,12], the challenge seems nearly insurmountable.

## 2. Another DNA revolution

Molecular barcodes, typically one or several small stretches of DNA, provide valuable characters to delineate species in all eukaryotic kingdoms of life. As such, standardized DNA regions were identified to supplement morphological identification: the mitochondrial cytochrome *c* oxidase 1 (COI) gene for animals [13], a combination of two chloroplast genes (matK + rbcl) for plants [14], the nuclear ribosomal internal transcribed spacer (ITS) for Fungi [15] and the universal 18S rRNA gene coupled with lineage-specific barcode genes in the highly diversified unicellular protists [16]. DNA barcoding has helped identify unrecognized taxa, and large databases of ‘DNA identifiers’ are now available in public databases (e.g. Barcode of Life Data Systems, BOLD [17]; the Protist Ribosomal Reference database, PR2 [18]). However, as communities typically comprise numerous small organisms with many rare species [19], Sanger sequence barcoding of individual organisms has proven inefficient for broad diversity surveys and impact assessments because of the time and money required.

In 2005, high-throughput sequencing (HTS) platforms became widely available, a technological revolution that now allows the detection of tens to hundreds of species simultaneously from whole-community samples in a matter of a few days. DNA is extracted from a collection of organisms (e.g. interstitial meiofauna, planktonic organisms or benthic sessile communities) or extracellular DNA (i.e. environmental DNA in water or sediments). Then a small fragment of a DNA marker gene is PCR amplified using general primers, yielding thousands of sequences per sample. DNA sequences are then sorted informatically, low-quality reads and contaminants are removed, and remaining sequences are clustered into molecular operational taxonomic units (OTUs). The resulting data can be compared with reference libraries of DNA barcodes to estimate richness and community composition [20].

This approach, also referred to as metabarcoding or metagenetics, was first applied to study bacterial and archaeal diversity [21], revealing up to 20 000 ‘species’ per litre of seawater [22]. It is also now a cost- and time-effective alternative for eukaryote community profiling [23,24]. In the past 10 years, HTS has been used to study benthic meiofauna diversity in shallow [25–27], deep-sea [28–30] and estuarine [31–33] sediments, macro- and meiofaunal diversity in seagrass beds [34] and oyster reefs [35], as well as planktonic diversity across the globe [36–43], particularly the diversity of picoplanktonic size fractions (less than 3 µm) [44–46]. The metabarcoding revolution has especially benefited our understanding of microscopic eukaryotic diversity—the unicellular (e.g. protist) or small multicellular (e.g. metazoan less than 1 mm) species that belong to some of the most challenging groups for taxonomists. These tiny organisms are highly abundant in marine environments and have long been recognized as functionally important in both benthic [47] and pelagic systems (e.g. the microbial loop [2]). However, little was known about their diversity patterns prior to using HTS. HTS has also been used to assess environmental impacts [48], understand trophic interactions [49–51] and track non-indigenous species [52].

## 3. What we have discovered in the ocean using metabarcoding

Studies using a metabarcoding approach have confirmed that diversity of eukaryotic organisms scales up with decreasing body size in both benthic and planktonic systems. Previously it was thought that intermediate-sized organisms held the most diversity [53–55], but earlier data were biased by our inability to distinguish the smallest species from each other using traditional methods. HTS-based studies directly comparing size-fractioned samples, either through sieving or filtration, highlight this new understanding. For example, Leray & Knowlton [35] found that two-thirds of the diversity on oyster reefs was smaller than 500 µm and Logares *et al*. [43] and De Vargas *et al*. [41] found a peak of diversity in the nanoplankton (3–20 µm) and pico-nanoplankton (0.8–5 µm), respectively. The smallest size fractions comprise unicellular taxa (protists) that constitute much of the phylogenetic diversity in the domain Eukaryota. The key contributions of these micro-eukaryotes to the nutrient cycling, primary productivity and trophic activity of marine ecosystems have long been recognized [56]. However, their diversity remained one of the least-known prior to DNA metabarcoding because most of these species lack diagnostic morphological features and cannot be cultured. HTS of samples collected directly in the environment has unveiled a staggering number of taxa within most supergroups of protists (e.g. Alveolata [41,57–59], Stramenopila [60,61], Excavata [41], Haptophyta [62], Rhizaria [28,41,63–65], Opisthokonta [66]), increasing known diversity by several orders of magnitude. Moreover, novel clades, some of them considered new kingdoms, keep being discovered (i.e. Picobiliphyta [67], Rappemonada [68]; reviewed in [69]).

Metabarcoding has been a powerful tool for quantifying the relative diversity of various taxonomic groups of microscopic metazoans in benthic systems. Nematodes, for example, are known to be the most abundant metazoans in marine sediments, with up to 20 million individuals per square metre, a density one order of magnitude higher than that of any other taxon [70]. They had also been considered to be one of the most diverse groups, with global estimates converging to one million species [71], in stark contrast with the limited number of marine nematode species that have been described to date (7152, WORMS [7]). Most nematodes lack clear homologous morphological characters, and DNA barcodes are often the only way to delineate species [72,73]. Metabarcoding analyses of shallow and deep-water sediments have confirmed their high contribution to total meiofaunal diversity in marine sediments [25,26,29,30,33,34,74]. However, several of these studies also showed for the first time an equal or higher representation of Platyhelminthes and Annelida at some sites [25,26,29,30,34]. High-energy sediments in intertidal zones are preferred habitats for Platyhelminthes, where they may represent up to 95% of the total meiofaunal biomass [75]. However, because their soft-body structure is altered by preservatives, they can only be studied alive or with light histological techniques [76]. Hence, they have mostly been disregarded in traditional surveys. Interstitial species of Annelida have been far less studied than their macrofaunal counterparts for similar reasons.

Similar analyses have broadened our understanding of the diversity of different pelagic groups. Because of their abundance, copepods are analogues of nematodes in pelagic systems. They represent important trophic links [77] and as such have been considered an important indicator of environmental changes. However, identifying copepods morphologically, a group with 11 181 described species and many more to describe [3], requires advanced taxonomic knowledge and time. Lower-level identifications are also problematic, with early life stages that are often misidentified or unidentified in monitoring programmes. As expected, the phylum Arthropoda is consistently the dominant metazoan group in metabarcoding analyses of plankton samples, with most OTUs belonging to copepods (i.e. 1009 out of 1554 in [37]; 5766 out of 7744 in [41]). Closely following Arthropoda, the phylum Chordata was particularly OTU-rich in the circumglobal analysis by De Vargas *et al*. [41] with 6795 OTUs (40% of total). A great majority of these were assigned to the genus *Doliolum* (6387 OTUs) potentially as a result of the lack of reference sequences among ascidians; however, if this pattern is confirmed (i.e. not the result of sequencing artefacts, pseudogenes or intra-individual polymorphism), this would mean that there are over 92 times more taxa within Ascidiacea than morphologically described species [41]. In the same study, Hydrozoa represented 86% (587 out of 682 OTUs) of the diversity of Cnidaria, with almost half (46%) remaining unassigned at lower taxonomic levels.

In addition to revealing an astounding number of unknown taxa, rapid and cost-effective community profiling of microscopic communities using HTS has provided a better understanding of the ecological factors influencing distribution patterns. Studies of meiofauna have been particularly interesting in this regard. With nearly 60% of all animal phyla having interstitial representatives, meiofaunal communities comprise a diversity of life-history traits and occupy a wide range of ecological niches. With the power of metabarcoding, some have argued that they represent an optimal ecological unit for effective biomonitoring of benthic habitats [33]. Analyses conducted in estuaries demonstrated the response of meiofaunal communities to differences in salinity, sediment particle sizes, oxygen, water flow, nutrient, pH and turbidity [31,33] with groups such as Nematoda and Plathyelminthes responding differently to some abiotic factors [33]. Interstitial metazoans also proved very sensitive to exposure to toxic oil; following the Deepwater Horizon catastrophe in the Gulf of Mexico, there was a pronounced and prolonged reduction of meiofaunal OTU richness, with a shift from metazoan to fungus-dominated interstitial assemblages [48]. Benthic foraminiferans represent another indicator of environmental perturbations because of their sensitivity to abiotic conditions and their short lifespans [78], but traditional surveys used time-consuming morphological identification of shells. Metabarcoding successfully quantified changes in the composition of foraminiferal communities in response to a gradient of sediment oxygenation and nutrient enrichment caused by salmon farming [79,80]. Molecular data also helped measure the impact of fish farms on communities of macro-invertebrates and provided comparable estimates of biotic indices to those obtained using morphological data alone [81], further confirming the promise of these tools for assessing the health status of marine systems [82]. As marine ecosystems are also increasingly threatened by invasive species, the taxonomic resolution of metabarcode data may prove to be a powerful early-warning tool for the detection of non-indigenous benthic species at egg or larval stages [42,52,83].

Finally, biogeographic patterns in microscopic groups have been unveiled with metabarcoding analyses that sidestep the taxonomic impediment. Available data have confirmed the previously proposed idea [84,85] that the most abundant taxa also often have wider distributions, providing strong evidence for a correlation between dispersal ability and other life-history traits. For example, Fonseca *et al*. [26] showed that the level of overlap in OTU composition of interstitial communities across a vast study area was highest among Nematoda and Platyhelminthes, the two most abundant phyla. Despite the presence of truly cosmopolitan taxa, groups of endemic OTUs were also identified as representative of biogeographic provinces [25,26,30]. Similarly, analysis of spatial patterns of planktonic diversity documented strong partitioning in plankton community composition that closely followed boundaries of water masses [40,41]. The first circumglobal metabarcoding analysis of ocean diversity [41] identified a significant correlation between geographical distance and community similarity at the scale of ocean basins for all size fractions of organisms living in the plankton. However, there were weaker levels of differentiation between communities of small-sized taxa than between communities of large sized taxa, suggesting increasing dispersal limitation with increasing body size.

## 4. Towards a global understanding of marine diversity?

Although trends are already emerging from individual studies, a global comparative dataset (i.e. Ocean Sampling Day [86]), integrating all kingdoms of life and all size classes, and bridging a range of marine ecosystems, would address outstanding questions in ecology beyond the most conspicuous species: do biodiversity patterns of unicellular eukaryotes and microscopic metazoans mirror in direction and magnitude the biodiversity patterns described for macrofauna (e.g. the latitudinal diversity gradient and the Coral Triangle biodiversity hotspot)? How do taxonomic, phylogenetic and functional diversity scale with geographical area, organism size and habitat diversity? How do patterns of commonness and rarity vary as a function of organism size and life-history traits? Does marine biodiversity vary predictably as a function of anthropogenic stress? Can we identify functional keystone groups that can be used as biological indicators? Answering these fundamental questions today is within reach but obstacles still remain (reviewed in [87]).

### (a) Methodological artefacts

The field of eukaryotic metabarcoding has been largely inspired by studies on prokaryotes for which analytical procedures have been extensively tested [88,89]. Studying eukaryotes presents its own technical challenges [87] that are now being addressed. Sources of variation in OTU detection and community profiling have been identified at each step of the metabarcoding analysis: sample collection [38], sample processing [90], PCR amplification (e.g. type of polymerase [91], target marker [34], primer set [30,36], primer selectivity [35,92]), sequencing (due to random sampling [93]), denoising of raw reads [94], chimera detection [95–97] and sequence clustering (e.g. type of algorithm [90,98], threshold level [26,30,43,99]). For example, pervasive amplification biases of some primers have led some to suggest caution when using OTU relative abundance in ecological assessments [92]. Others have shown that presence–absence data are also sensitive to biases because random processes affect the reproducibility of rare OTUs [93]. In addition to methodological concerns, pseudogenes and intra-individual polymorphisms [100] can lead to inflated diversity metrics by increasing the number of predicted OTUs. The optimization of protocols and the use of controls (i.e. mock communities, technical replicates) can help increase the reliability of metabarcoding for a range of applications. Overall, there is growing evidence that methodological artefacts need to be carefully considered for the design and interpretation of studies using metabarcoding.

### (b) Lack of standardization of target markers

**Figure 1.**
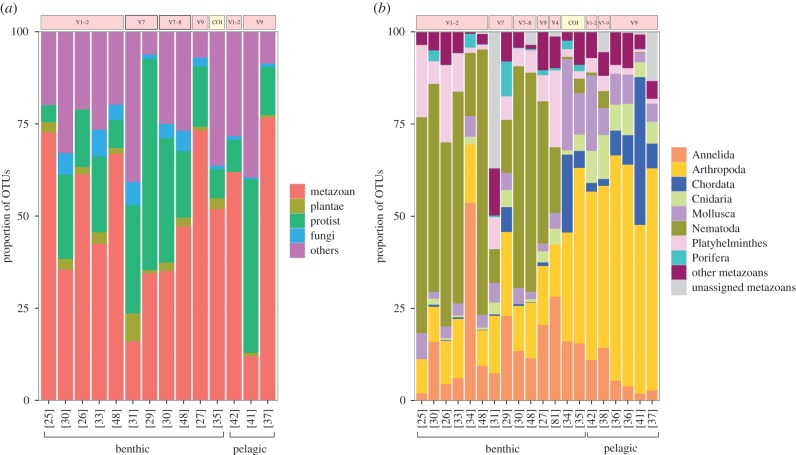
Proportion of OTUs among major groups of organisms (*a*) and among metazoan phyla (*b*) reported in studies characterizing benthic and planktonic communities using metabarcoding. Studies that strictly looked at protist diversity are not represented. The marker region targeted in each study (COI or 18S variable region) is indicated. Note that several studies targeted multiple markers. The category ‘Others’ in panel (*a*) comprises OTUs that were reported as unassigned, environmental sequences or prokaryotes. The category ‘other metazoans’ in (*b*) comprises the following phyla: Acanthocephala, Brachiopoda, Bryozoa, Cephaloryncha, Chaetognatha, Ctenophora, Cycliophora, Dicyemida, Echinodermata, Entoprocta, Gastrotricha, Gnathostomulida, Hemichordata, Micrognathozoa, Nemertea, Orthonectida, Phoronida, Placozoa, Rotifera, Sipuncula, Tardigrada and Xenacoelomorpha.

The use of different target fragments between independent studies represents the principal limitation to addressing regional scale and long-term questions in biodiversity because amplicon data obtained from non-homologous regions cannot be compared. Most published studies using broad-range primers ([25–38,41,42,48,81], omitting studies strictly looking at protists) target hypervariable regions of the 18S rRNA gene (94%), with the scientific community now slowly converging towards the use of the V1–2 and V9 (hyper)variable regions; these were amplified in 39% and 33% of studies, respectively (figure 1*a*,*b*). The prevalence of studies targeting 18S regions largely stems from the versatility of PCR primers that represent convenient tools to screen the entire eukaryotic domain (figure 1*a*). By contrast, the mitochondrial COI gene, the standard barcoding gene for animals, has been targeted in only 11% of studies.

### (c) Lack of taxonomic resolution

Primer versatility comes at the price of taxonomic resolution. There is now ample evidence that all 18S rRNA regions greatly underestimate the true number of metazoan species, the functional units of marine ecosystems. For example, Tang *et al*. [101] showed that 18S reduced estimates of the diversity of microscopic interstitial metazoans by a factor of 0.4 relative to morphology, whereas COI, which efficiently identified cryptic lineages, increased diversity estimates by a factor of 7.6. Similarly, Wu *et al*. [102] identified the V9 region as the most informative for taxonomic classification of copepods, but levels of nucleotide variation enabled clear differentiation only between genera and sometimes species within a few taxonomic groups. Mohrbeck *et al*. [103] came to the same conclusion after evaluating the effectiveness of the V1–V2 region for planktonic communities after identifying strictly identical sequences (100% similarity) shared by confamiliar species. The performance of 18S for delineating protist species has been more challenging to evaluate because species boundaries have seldom been confirmed within an integrative taxonomic framework (i.e. morphology, ultrastructure and DNA taxonomy of cultured strains [104]). However, evidence from whole-genome sequences suggests similar limitations for biodiversity surveys [105].

**Figure 2.**
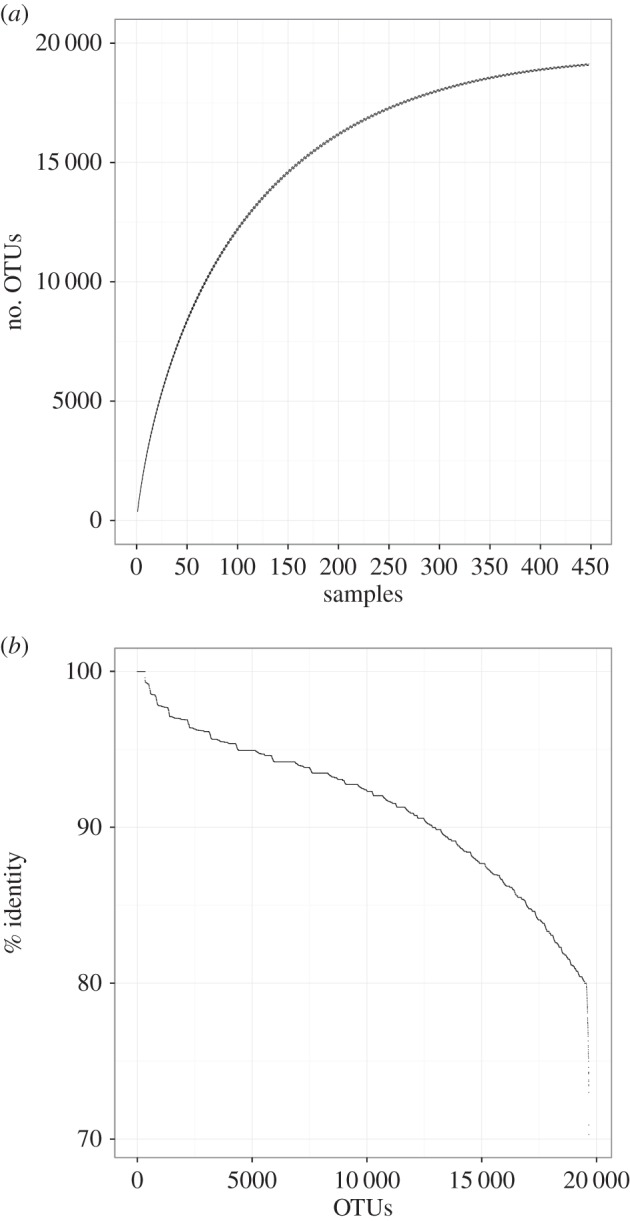
Metazoan diversity detected in the plankton based on 18S V9 sequencing. Sample-based rarefaction ((*a*), without replacement) and levels of sequence similarity to reference barcodes of the PR2 database (*b*) [18] were calculated using a dataset combining OTUs detected during the Tara Ocean circumglobal expedition [41] and OTUs collected in surface water around coral reefs [36] and along a depth profile [37] in the Red Sea. Together, these studies account for most of the sampling and sequencing effort conducted in the ocean for metazoans, with 448 samples and approximately 258 million metazoan sequences. OTU-representative sequences of each study were clustered using SWARM (*d* = 1) [106].

To further illustrate the limitations of 18S for censusing metazoan marine diversity, we combined V9 datasets of the Tara Ocean circumglobal expedition [41] with plankton community profiles for surface water around coral reefs [36] and along a depth profile [37] in the Red Sea. Together, these studies account for most of the sampling and sequencing effort conducted in the ocean for metazoans, with 448 samples and approximately 258 million metazoan sequences. After clustering OTU-representative sequences of respective studies using Swarm (*d* = 1) [106], there were a total of 19 671 metazoan OTUs, which represents only a tiny fraction of what is known to live in the ocean (table 1). Yet, the rate of discovery of metazoan OTUs with the V9 region of the 18S rRNA gene is beginning to plateau after 448 samples (figure 2*a*). While this may be partly caused by the highly conservative quality control applied to the Tara dataset (e.g. removal of all unique reads present in a single sample) and the fact that some marine metazoans are unlikely to be regularly present in plankton samples, this pattern is also probably driven by low levels of variability of the V9 region.

**Table 1.** Total number of accepted metazoan species in the ocean according to World Register of Marine Species (WORMS) compared with the diversity of 18S V9 OTUs detected in metabarcoding analyses of plankton samples collected circumglobally.

	accepted marine species in WORMS	18S V9 OTUs in plankton^a^
Acanthocephala	468	0
Annelida	12 862	404 (48)
Arthropoda	57 871	9497 (205)
Brachiopoda	396	8 (4)
Bryozoa	6167	15 (5)
Cephalorhyncha	238	13
Chaetognatha	131	531 (7)
Chordata	22 248	6972 (32)
Cnidaria	11 407	842 (82)
Ctenophora	192	33 (9)
Cycliophora	2	0
Dicyemida	122	1
Echinodermata	7252	74 (19)
Entoprocta	180	12
Gastrotricha	497	2
Gnathostomulida	98	0
Hemichordata	130	12 (3)
Mollusca	45 219	560 (89)
Nematoda	7152	30 (6)
Nemertea	1359	81 (4)
Orthonectida	25	0
Phoronida	19	0
Placozoa	1	0
Platyhelminthes	12 230	148 (12)
Porifera	8476	25 (4)
Rotifera	187	7 (2)
Sipuncula	147	0
Tardigrada	193	2
Xenacoelomorpha	433	0
unassigned metazoan	n.a.	402
total	195 702	19 671 (531)

^a^De Vargas *et al*. [41] and Pearman *et al*. [36,37] for a total of 448 plankton samples and approximately 258 million reads. OTU-representative sequences of each study were clustered using SWARM [106] (*d* = 1). Numbers between parentheses indicate OTUs with identity greater than 99% to the V9 PR2 database [18]. Note that some metazoans are unlikely to be routinely present in plankton samples as either adults or larvae.

### (d) Paucity of reference barcode data in public databases

While representative barcode sequences are available across most of the branches of the tree of life (however, see [16] for protists), the lack of coverage at lower taxonomic levels currently hinders interpretation of metabarcoding data. Most OTUs remain identified to taxonomic groups that have limited taxonomic and functional relevance in biodiversity inventories, ecological studies or monitoring initiatives. For example, very few metazoan OTUs in the 18S V9 circumglobal plankton dataset matched sequences of the Protist Ribosomal Reference (PR2 [18]) database with levels of sequence similarity typical of genus-level matches (2.7% with more than 99% similarity, figure 2*b* and table 1) (see [102] for threshold justification). This problem is also acute in studies targeting the hypervariable COI marker because COI tends to saturate at higher taxonomic levels. High levels of homoplasy between distant phylogenetic groups decrease the likelihood of confidently assigning OTUs in the absence of close representatives at lower taxonomic levels. This translated into a large number of OTUs classified as ‘unidentified’ in a recent benthic survey (28.3% [35]). Ongoing efforts by various working groups of the Consortium for the Barcode of Life (CBOL, www.barcodeoflife.org) to populate public barcode repositories and compile available data into curated databases (i.e. PR2 [18], Silva [107]) will help fill this gap.

## 5. Concluding remarks

The trade-off between amplification success and taxonomic resolution has long been recognized in PCR-based studies. Markers with highly conserved flanking regions underestimate the true number of species, whereas markers with more variable flanking regions usually provide better estimates of species richness. Shotgun metagenomics and metatranscriptomics remove the need to compromise, but they require a much higher sequencing depth per sample and have proved less effective at detecting rare taxa [108]. As a result, there is an urgent need to synergize and coordinate metabarcoding efforts because metabarcoding is likely to remain one of the prime methods for biodiversity monitoring and ecological studies for at least a few more years. For example, a strategy in which samples would be analysed using a two-step approach combining gene regions that vary in taxonomic coverage and taxonomic resolution would better reflect marine diversity and enable future comparative studies. A first step could include profiling whole eukaryotic communities at a coarse taxonomic level by targeting 18S rRNA V1–V2 or V9 regions as a ‘pre-metabarcode’ (cf. [16]), two regions for which highly versatile primers are available (see [109] for *in silico* tests of existing primer sets). A second step could target one or several more variable markers with broad-range lineage-specific primers (i.e. COI [50] and 12S [110] for metazoans, ITS for fungi [15]) to provide enhanced level of detail for groups that remain under-explored. Importantly, environmental vouchers (e.g. DNA and RNA extracts, remaining tissue homogenates) are as important for biodiversity studies as individual vouchers are for taxonomy. Therefore, we may also envision the creation of a network of museum-based repositories from which environmental vouchers could be loaned for complementary metabarcoding or genomics applications.

The ocean represents approximately 70% and more than 90% of the Earth's surface and habitable volume, respectively. A holistic understanding of marine diversity will only become possible with coordinated efforts, further methodological developments, strict methodological standards and consistency of experimental designs [111]. Predicting how species respond to environmental change (e.g. range expansion and extinction) will only be possible if we include markers in metabarcoding analyses that are both consistent across taxa and hypervariable. Yet the need for biodiversity baselines cannot be postponed in this rapidly changing world, and today's methods for metabarcoding are already very powerful. Thus, it is also important, as Voltaire noted, not to let the perfect become the enemy of the good.
